# Mitochondrial Dysfunction in Cardiomyopathy and Heart Failure: From Energetic Collapse to Therapeutic Opportunity

**DOI:** 10.3390/biom15111572

**Published:** 2025-11-09

**Authors:** Nikola Pavlović, Petar Todorović, Mirko Maglica, Marko Kumrić, Katarina Vukojević, Zenon Pogorelić, Joško Božić

**Affiliations:** 1 Department of Pathophysiology, University of Split School of Medicine, 21000 Split, Croatia; 2 Laboratory for Cardiometabolic Research, University of Split School of Medicine, 21000 Split, Croatia; 3 Department of Anatomy, Histology and Embryology, University of Split School of Medicine, 21000 Split, Croatia; 4 Department of Anatomy, School of Medicine, University of Mostar, 88000 Mostar, Bosnia and Herzegovina; 5 Department of Otorhinolaryngology, University Hospital of Split, 21000 Split, Croatia; 6 Mediterranean Institute for Life Sciences, University of Split, 21000 Split, Croatia; 7 Center for Translational Research in Biomedicine, University of Split School of Medicine, 21000 Split, Croatia; 8 Department of Pediatric Surgery, University Hospital of Split, 21000 Split, Croatia; 9 Department of Surgery, School of Medicine, University of Split, 21000 Split, Croatia

**Keywords:** mitochondrial dysfunction, cardiomyopathy, heart failure, bioenergetics, mitochondrial dynamics

## Abstract

The heart’s relentless contractile activity depends critically on mitochondrial function to meet its extraordinary bioenergetic demands. Mitochondria, through oxidative phosphorylation, not only supply ATP but also regulate metabolism, calcium homeostasis, and apoptotic signaling, ensuring cardiomyocyte viability and cardiac function. Mitochondrial dysfunction is a hallmark of cardiomyopathies and heart failure, characterized by impaired oxidative phosphorylation, excessive production of reactive oxygen species (ROS), dysregulated calcium handling, and disturbances in mitochondrial dynamics and mitophagy. These defects culminate in energetic insufficiency, cellular injury, and cardiomyocyte death, driving heart disease progression. Diverse cardiomyopathy phenotypes exhibit distinct mitochondrial pathologies, from acute ischemia-induced mitochondrial collapse to chronic remodeling seen in dilated, hypertrophic, restrictive, and primary mitochondrial cardiomyopathies. Mitochondria also orchestrate cell death and inflammatory pathways that worsen cardiac dysfunction. Therapeutic strategies targeting mitochondrial dysfunction, including antioxidants, modulators of mitochondrial biogenesis, metabolic therapies, and innovative approaches such as mitochondrial transplantation, show promise but face challenges in clinical translation. Advances in biomarker discovery and personalized medicine approaches hold promise for optimizing mitochondrial-targeted therapies. Unlike previous reviews that examined these pathways or interventions individually, this work summarizes insights into mechanisms with emerging therapeutic strategies, such as SGLT2 inhibition in HFpEF, NAD^+^ repletion, mitochondrial transplantation, and biomarker-driven precision medicine, into a unified synthesis. This framework underscores the novel contribution of linking basic mitochondrial biology to translational and clinical opportunities in cardiomyopathy and heart failure. This review synthesizes the current understanding of mitochondrial biology in cardiac health and disease, delineates the molecular mechanisms underpinning mitochondrial dysfunction in cardiomyopathy and heart failure, and explores emerging therapeutic avenues aimed at restoring mitochondrial integrity and improving clinical outcomes in cardiac patients.

## 1. Introduction

The heart is an extraordinary organ that works incessantly throughout life, and is dependent on a continuous supply of energy to maintain its contractile activity and ensure haemodynamic stability [1,2,3,4,5]. This immense energy demand is primarily met by the mitochondria, the cell organelles known as the powerhouse of the cell [6,7,8]. In healthy heart muscle, the mitochondria are organised in such a way that they meet the high bioenergetic demands by producing adenosine triphosphate (ATP) through oxidative phosphorylation. These organelles not only generate energy but also play a crucial role in regulating cellular metabolism, calcium homeostasis, and apoptotic signalling, thus maintaining cardiomyocyte viability and overall cardiac function [1,9,10,11,12].

The fact that mitochondria make up over 30% of the volume of cardiomyocytes highlights how essential they are to heart physiology [13]. Under normal circumstances, fatty acids are the primary substrate for the adult heart’s nearly entirely aerobic metabolism, which is augmented by the oxidation of glucose and lactate. The heart can effectively adjust to changing energy needs thanks to its metabolic flexibility [14,15,16]. As electrons go through several complexes connected to proton pumping and chemiosmotic ATP synthesis, the electron transport chain (ETC), which is enmeshed in the inner mitochondrial membrane, propels ATP production [17,18]. The heart’s ability to maintain contractile action can be significantly impacted by any disruption in these precisely regulated mechanisms [8,19].

Beyond energy generation, mitochondrial dynamics, including the processes of biogenesis, fission, fusion, and mitophagy, are crucial for maintaining a healthy mitochondrial population [20,21]. Mitochondrial biogenesis is governed by transcription factors and coactivators such as PGC-1α, which coordinate the expression of nuclear and mitochondrial genes responsible for mitochondrial replication and function [22]. Mitochondrial fission and fusion enable the organelles to adapt to metabolic needs and facilitate the removal or repair of damaged regions [21]. Mitophagy, a selective form of autophagy, ensures the quality control of mitochondria by degrading dysfunctional mitochondria to prevent the accumulation of oxidative damage and maintain cellular homeostasis. Collectively, these mitochondrial quality control mechanisms are vital for the preservation of cardiac function, particularly under physiological stress [23,24].

**Figure 1:**
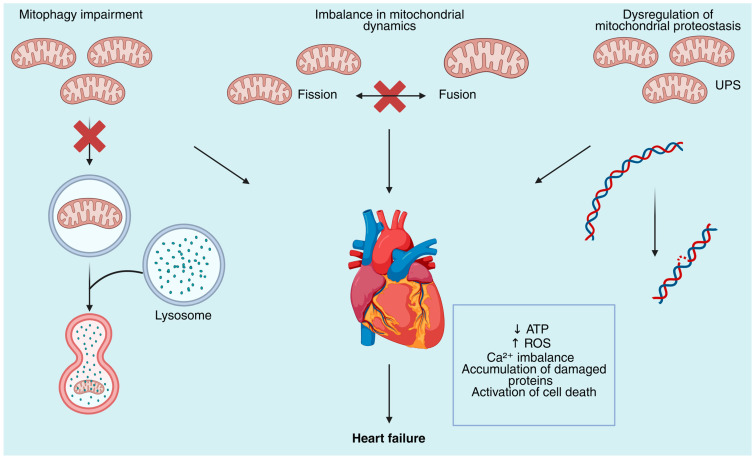
Mitochondrial quality control defects contribute to the development of heart failure. Mitochondrial dysfunction in cardiomyocytes arises from defects in multiple quality control pathways. Impaired mitophagy leads to the accumulation of dysfunctional mitochondria due to the inability of lysosomal degradation to occur. Imbalance in mitochondrial dynamics disrupts the fission–fusion cycle required for mitochondrial remodeling and adaptation, leading to altered morphology and bioenergetic failure. Dysregulation of mitochondrial proteostasis, including defective ubiquitin–proteasome system (UPS) activity, promotes accumulation of damaged proteins and mitochondrial DNA (mtDNA) instability. Collectively, these defects result in decreased ATP production, excessive generation of reactive oxygen species (ROS), calcium (Ca^2+^) dysregulation, proteotoxic stress, and activation of cell death pathways, ultimately contributing to the progression of heart failure.

The heart’s reliance on mitochondrial integrity becomes glaringly evident in pathological states such as cardiomyopathy and heart failure, where mitochondrial dysfunction emerges as a central contributor to disease progression [25]. Cardiomyopathies encompass a heterogeneous group of myocardial disorders that impair cardiac structure and function, often culminating in heart failure, a leading cause of morbidity and mortality worldwide [26,27]. In these conditions, mitochondrial defects manifest as impaired oxidative phosphorylation, excessive reactive oxygen species (ROS) production, disrupted calcium handling, and imbalanced mitochondrial dynamics, which collectively drive energetic insufficiency and cellular damage (Figure 1) [28,29].

Energy deprivation in the myocardium initiates a downward spiral of contractile failure and adverse remodeling [3,30]. Moreover, mitochondria-derived signals can trigger cell death pathways and inflammatory cascades that exacerbate cardiac injury and promote disease progression. Thus, understanding mitochondrial biology in the context of cardiac health and disease is essential for the development of novel therapeutic strategies aimed at preserving or restoring mitochondrial function [31,32,33].

Although numerous reviews have examined individual aspects of mitochondrial biology in cardiovascular disease [7,8], few have provided an integrated summary that spans mechanistic insights, diverse cardiomyopathy phenotypes, and emerging therapeutic approaches. Prior work has typically focused on oxidative stress, substrate metabolism, or single therapeutic strategies in isolation. By contrast, the present review unifies recent advances, including SGLT2 inhibition in HFpEF, NAD^+^ repletion, mitochondrial transplantation, and biomarker-driven precision medicine, within the broader framework of mitochondrial dysfunction across cardiomyopathy subtypes. In doing so, it highlights both mechanistic commonalities and disease-specific vulnerabilities, offering a novel perspective that bridges basic mitochondrial biology with translational and clinical opportunities in heart failure. This review aims to provide a comprehensive overview of mitochondrial dysfunction in cardiomyopathy and heart failure, outlining the underlying molecular mechanisms and emphasizing emerging therapeutic opportunities. We begin by thoroughly examining mitochondrial function in a healthy heart, laying the groundwork for later sections that explore pathophysiological changes, various cardiomyopathy phenotypes, mitochondria-driven cell death, and the potential for clinical applications of mitochondrial-targeted interventions.

## 2. Mechanisms of Mitochondrial Dysfunction in Heart Failure

Mitochondrial dysfunction is a hallmark of cardiomyopathies and heart failure. It results from a complex interplay of molecular mechanisms that impair the energy production of the heart muscle and cellular homeostasis [34,35]. An important factor that sets this pathological cascade in motion is oxidative stress, which leads to damage to the mitochondrial DNA (mtDNA). The failing heart is characterised by an increased production of reactive oxygen species (ROS), which the mitochondrial ETC mainly generates [36]. In contrast to antioxidant enzymes, whose activities are relatively preserved, excessive mitochondrial ROS increasingly damages the mtDNA, which codes for essential subunits of the respiratory complexes. This leads to a vicious cycle in which the damaged mtDNA impairs mitochondrial function, further increasing ROS production and perpetuating cellular damage. ROS also initiates maladaptive myocardial remodeling by activating matrix metalloproteinases responsible for extracellular matrix degradation, promoting myocyte hypertrophy, apoptosis, and interstitial fibrosis, key features of progressive heart failure [37,38,39].

Beyond oxidative damage, mitochondrial dysfunction in cardiomyopathy is closely linked to impaired oxidative phosphorylation (OXPHOS) and consequent ATP depletion. Defects in substrate oxidation and the electron transport chain, affecting complexes I through V, lead to decreased bioenergetic capacity in failing myocardium [40,41,42]. The defects interrupt the cardiac phosphocreatine/ATP energy shuttle, leading to elevated levels of ADP that hinder contractile processes. Research consistently shows reduced oxidative phosphorylation (OXPHOS) activity in both human and animal models of heart failure, highlighting the mitochondrion as a key site of energy deficiency that contributes to contractile dysfunction [43,44,45]. Mitochondrial calcium overload further exacerbates OXPHOS dysfunction. Calcium regulates key dehydrogenases within the tricarboxylic acid cycle; however, excessive mitochondrial calcium accumulation triggers opening of the mitochondrial permeability transition pore (mPTP), dissipating membrane potential and collapsing ATP synthesis capability [46,47,48].

The regulation of mitochondrial calcium homeostasis is integral to cardiac function, involving calcium uptake primarily through the mitochondrial calcium uniporter (MCU) and efflux via Na^+^/Ca^2+^ exchangers. In cardiomyopathy and heart failure, dysregulated calcium flux disrupts this delicate balance, favoring the opening of mPTP and subsequent cell death pathways. The mPTP is a high-conductance channel in the inner mitochondrial membrane, whose pathological opening not only depolarizes mitochondria but also releases pro-apoptotic factors such as cytochrome c, intensifying cardiomyocyte loss and contractile impairment [49,50,51].

At the same time, abnormalities in mitochondrial quality control mechanisms exacerbate cardiac mitochondrial dysfunction. Mitochondrial fission and fusion, a dynamic process essential for adapting mitochondrial morphology and function, become dysregulated in cardiomyopathic hearts. Impairments in fusion proteins (e.g., Mitofusins 1 and 2) and fission proteins (e.g., Drp1) disrupt mitochondrial network integrity and prevent segregation of damaged mitochondrial segments. This imbalance leads to the accumulation of dysfunctional mitochondria with reduced oxidative capacity and enhanced ROS release, further impairing cardiomyocyte function [52,53,54].

Mitophagy, the selective autophagic clearance of damaged mitochondria, is another critical facet of mitochondrial quality control negatively affected in heart failure. Deficient activation of mitophagy pathways, involving key regulators such as PINK1 and Parkin, results in the accumulation of structurally abnormal and bioenergetically compromised mitochondria [55,56,57]. This failure to clear damaged mitochondria intensifies oxidative stress and energetic deficits, accelerating cardiomyocyte dysfunction and death. Moreover, emerging evidence indicates that mitophagy and mitochondrial dynamics are tightly coordinated processes; disruption in one often impairs the other, creating a feedback loop that compromises mitochondrial homeostasis in failing hearts [58,59,60].

**Figure 2:**
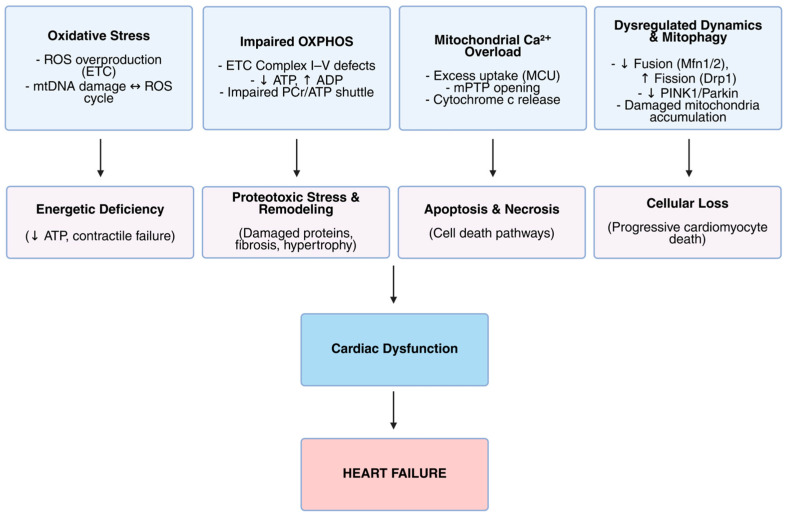
Mitochondrial dysfunction and its role in the pathogenesis of heart failure. This schematic illustrates key mitochondrial abnormalities that contribute to the progression of heart failure. The top row identifies four primary mitochondrial dysfunctions: oxidative stress characterized by reactive oxygen species (ROS) overproduction and mitochondrial DNA (mtDNA) damage; impaired oxidative phosphorylation (OXPHOS) involving defects in the electron transport chain (ETC) leading to reduced ATP production and disrupted energy shuttle; mitochondrial calcium overload resulting from excessive uptake (MCU) and mitochondrial permeability transition pore (mPTP) opening, causing cytochrome c release; and dysregulated mitochondrial dynamics and mitophagy, including decreased fusion, increased fission, downregulation of PINK1/Parkin pathways, and accumulation of damaged mitochondria. These disturbances trigger downstream pathological processes such as energetic deficiency, proteotoxic stress with remodeling, apoptosis and necrosis, and cellular loss. Together, these mechanisms culminate in cardiac dysfunction, ultimately leading to heart failure.

In summary, mitochondrial dysfunction in cardiomyopathy and heart failure arises from intertwined mechanisms: oxidative stress induces mtDNA damage and excessive ROS; impaired OXPHOS reduces ATP synthesis; disturbed calcium homeostasis leads to pathological mPTP opening; and dysregulated mitochondrial dynamics and deficient mitophagy degrade mitochondrial quality. These mechanisms are summarized schematically in Figure 2, illustrating their contribution to energetic deficits, cellular injury, and ultimately cardiac dysfunction leading to heart failure. Together, these molecular perturbations precipitate the energetic collapse and cellular damage characteristic of heart failure, highlighting the mitochondrion as a central nexus and promising therapeutic target in cardiac disease.

## 3. Mitochondrial Dysfunction Across Different Types of Cardiomyopathy

Mitochondrial dysfunction is a common axis in cardiomyopathies, but its mode and consequences vary by subtype, informing prognosis and therapy [61]. Mitochondrial energetic failure integrates deranged substrate utilization, impaired oxidative phosphorylation (OXPHOS), and redox stress, which cumulatively depresses ATP supply [61].

Although ischemic cardiomyopathy is not traditionally classified as a primary cardiomyopathy, since it is secondary to coronary artery disease, this heart failure phenotype exhibits a number of distinct mitochondrial characteristics. In ischemic cardiomyopathy, ischemia–reperfusion (I/R) triggers a cascade of mitochondrial calcium overload, reactive oxygen species (ROS) bursts, and the opening of the mPTP, ultimately driving necrosis and apoptosis [62]. During ischemia, fatty acid oxidation (FAO) is curtailed, and glucose oxidation becomes relatively favored, a shift that can be therapeutically exploited [63]. Pharmacologic FAO inhibition (e.g., malonyl-CoA decarboxylase inhibition) increases glucose oxidation and limits ischemic injury in preclinical models, highlighting metabolic control as a key component of cardioprotection [63]. Consistent with this, post-ischemic suppression of FAO promotes regeneration and functional recovery in mammalian hearts, linking substrate choice to repair capacity [64]. In contrast, non-ischemic etiologies typically impose chronic mitochondrial stress rather than an acute energetic collapse, as seen in viral myocarditis, where viral replication and innate immune signaling disrupt mitochondrial dynamics, bioenergetics, and antiviral response pathways, fostering progression to dilated phenotypes [65,66].

Human dilated cardiomyopathy (DCM) myocardium displays fragmented mitochondrial networks, cristae disorganization, and reduced respiratory chain capacity, indicating profound ultrastructural and functional remodeling [67]. Mitochondrial cristae defects in failing human hearts correlate with impaired OXPHOS efficiency and heightened susceptibility to cell death, placing inner-membrane architecture at the center of disease mechanisms [68]. In parallel, lipotoxicity from inefficient FAO and toxic lipid intermediates further impairs mitochondrial function, amplifies oxidative stress, and worsens ventricular dilation and failure [69]. Together, architectural remodeling and metabolic derangements converge on energetic failure in DCM [67,68,69].

In hypertrophic cardiomyopathy (HCM), emerging evidence underscores mitochondrial dysfunction as a key contributor to disease pathology. Mutations in sarcomeric proteins increase the ATP cost of contraction, termed “mechano-energetic uncoupling”, creating an intrinsic energetic inefficiency even at rest [70,71,72,73]. Phosphorus magnetic resonance spectroscopy has consistently revealed reduced phosphocreatine/ATP ratios in HCM patients, which correlate with diastolic impairment and heightened arrhythmic risk [72,73]. Recent analyses of explanted HCM hearts have revealed structural disorganization of mitochondrial networks and uncoupling of respiration from NADH supply. Importantly, NADH-driven respiration could be rescued ex vivo, underscoring both the centrality and therapeutic tractability of mitochondrial dysfunction in HCM [74]. These observations are supported by studies, such as the feline HCM model by Christiansen et al., which directly demonstrated that mitochondrial OXPHOS capacity is impaired, accompanied by increased oxidative stress, establishing energy depletion as a fundamental aspect of HCM pathophysiology that likely contributes to diastolic dysfunction, arrhythmia risk, and progression to heart failure [75].

Restrictive cardiomyopathy (RCM) often arises from infiltrative disorders—most prominently cardiac amyloidosis and storage diseases such as Fabry, where diastolic dysfunction occurs with relatively preserved systolic function early on [76]. In amyloidosis, mitochondrial dysfunction is typically secondary to proteotoxic stress from amyloid species. Cardiotropic light chains and transthyretin (TTR) assemblies trigger oxidative injury and disrupt Ca^2+^ handling, impairing respiration and contractile performance [77,78,79]. Experimental work shows that amyloidogenic light chains directly provoke oxidant stress, contractile dysfunction, and apoptosis in cardiomyocytes via a non-canonical p38α MAPK pathway, supporting a causal link between proteotoxic aggregates and mitochondrial/energetic failure [78]. Complementary studies demonstrate metabolic (bioenergetic) dysfunction in human cardiomyocytes exposed to light-chain fibrils [80]. In TTR amyloidosis, cardiomyocytes exposed to TTR fibrils exhibit decreased force production and prolonged Ca^2+^ transients, indicating electrophysiologic–energetic coupling defects [77], while recent reviews synthesize evidence that oxidative stress, impaired mitochondrial function, and Ca^2+^ dysregulation are central to ATTR-CM pathogenesis [81]. Beyond amyloidosis, Fabry disease illustrates how lysosomal sphingolipid accumulation perturbs autophagy and secondarily injures mitochondria, contributing to myocardial remodeling and conduction/arrhythmic vulnerability [82,83].

Primary mitochondrial cardiomyopathies establish a direct causal link between mitochondrial dysfunction and cardiac disease [29]. The common mtDNA mutation m.3243A>G, classically associated with MELAS, can manifest as either hypertrophic or dilated cardiomyopathy depending on heteroplasmy levels and tissue distribution [84]. Functional studies using patient-derived iPSC-cardiomyocytes with high m.3243A>G loads demonstrate impaired mitochondrial respiration in all cases, but only cells from patients with clinical cardiomyopathy exhibited reduced ATP levels and disrupted calcium signaling, emphasizing respiratory chain dysfunction and metabolic heterogeneity as direct effects of the mutation [85]. Additional mtDNA variants continue to be identified; for instance, the m.4300A>G mutation in MT-TI (tRNA Ile) has been reported in hypertrophic cardiomyopathy [86]. Nuclear gene defects in mtDNA maintenance, such as mutations in TK2 and POLG, can result in mtDNA depletion or multiple deletions, undermining respiratory chain integrity and precipitating severe early-onset cardiomyopathy [87]. Barth syndrome, resulting from TAFAZZIN mutations and defective cardiolipin remodeling, destabilizes respiratory supercomplexes and disrupts cristae architecture, producing dilated or non-compaction cardiomyopathy with high risk of heart failure [88].

Collectively, ischemic cardiomyopathy represents an acute mitochondrial collapse driven by Ca^2+^ overload, ROS bursts, and mPTP opening [62,63,64], whereas DCM is dominated by chronic cristae remodeling and lipotoxic stress [67,68,69]. HCM is marked by mechano-energetic uncoupling, impaired PCr/ATP transfer, and OXPHOS inefficiency [70,71,72,73], while RCM largely reflects secondary mitochondrial injury imposed by proteotoxic amyloid species or lysosomal storage pathology [76,77,78,79,80]. Genetic mitochondrial cardiomyopathies firmly establish causality, as mtDNA point mutations (e.g., m.3243A > G), defects in mtDNA maintenance (TK2, POLG), and TAFAZZIN-related cardiolipin remodeling produce cardiomyopathy phenotypes, highlighting opportunities for precision therapies such as metabolic modulation, nucleoside supplementation, or cardiolipin stabilization [29,84,85,86,87,88].

In addition to the traditional energetic and structural changes observed in various cardiomyopathy subtypes, emerging research underscores the importance of cytoskeletal interactions and mitochondrial diversity in influencing cardiomyocyte performance and susceptibility. Incorporating these elements yields a fuller picture of mitochondrial disease mechanisms and could uncover novel avenues for precision therapies.

## 4. Mitochondrial-Driven Cell Death Pathways in Heart Failure Progression

Mitochondria play a central role in regulated cardiomyocyte death, orchestrating multiple cell-death modalities and therefore contributing to heart failure progression [1,41]. In failing myocardium, both apoptotic and non-apoptotic pathways converge on mitochondrial dysfunction, resulting in energy collapse and loss of viable cardiomyocytes [41,89]. Dysregulated mitochondria also act as initiators of inflammatory signaling via the release of damage-associated molecular patterns (DAMPs), amplifying cardiomyocyte injury [90,91].

Cardiomyocyte apoptosis in heart failure is predominantly executed through the intrinsic, mitochondria-dependent pathway. Pro-apoptotic BAX and BAK oligomerize in the outer mitochondrial membrane, inducing permeabilization and cytochrome c release [92,93,94]. In the cytosol, cytochrome c interacts with Apaf-1 and ATP to assemble the apoptosome, activating initiator caspase-9, followed by caspase-3, ultimately driving controlled cellular disintegration [95]. SMAC/DIABLO release further neutralizes IAPs (inhibitors of apoptosis proteins), amplifying caspase activation [92]. In experimental rodent models and human failing hearts, increased BAX expression, mitochondrial cristae remodeling, and elevated caspase activity strongly correlate with myocardial dysfunction [96,97]. Under acute stress (e.g., ischemia, reperfusion, or severe oxidative stress), opening of the mPTP collapses mitochondrial membrane potential, halting ATP synthesis and triggering osmotic swelling [98]. Outer membrane rupture releases pro-death-factors and ROS, leading to regulated necrotic cell death [99]. In parallel, necroptosis—mediated by RIPK1, RIPK3, and MLKL, has emerged as a genetically programmed necrotic pathway that frequently intersects with mitochondrial dysfunction [100]. In models of ischemia–reperfusion injury, inhibition of Cyclophilin D (an mPTP regulator) reduces necrosis, while blockade of RIPK3/MLKL attenuates infarct size and preserves cardiac function [101]. Thus, mitochondria-driven necrotic signaling critically contributes to cardiomyocyte loss. Importantly, apoptotic and necrotic pathways do not operate in isolation within cardiomyocytes. Emerging evidence suggests that they are interconnected through mitochondrial dysfunction, particularly via the sub-threshold opening of the mPTP. When the mPTP is low or partially open, it can trigger apoptotic signaling if ATP levels remain sufficient. However, if ATP levels fall severely or caspase activity is inhibited, the same stimulus may result in necrotic cell death instead [102]. This continuum between apoptosis and necrosis is described by the concept of “necroptosis”, where the fate of the cell hinges on cellular energy status and caspase activation. Meanwhile, mitochondrial dysfunction leads to the release of DAMPs into the cytosol and extracellular space. These include mtDNA fragments, ROS, oxidized phospholipids such as cardiolipin, and mitochondrial peptides. Once released, these molecules engage innate immune sensors, such as TLR9, NLRP3 inflammasome, cGAS-STING, and TLR4, thus amplifying local inflammatory cascades [103,104]. Persistent mitochondrial-driven cell loss undermines myocardial integrity and contractility, establishing a vicious cycle that ultimately contributes to heart failure progression. Therapeutic strategies targeting components of these pathways hold promise: inhibiting BAX/BAK activation to prevent cytochrome c release, Cyclophilin D antagonists to block mPTP opening, and RIPK1/RIPK3/MLKL inhibitors to reduce necroptosis may preserve cardiomyocyte viability. Such approaches, by attenuating both energetic collapse and inflammatory amplification, could slow or reverse adverse remodeling in heart failure.

## 5. Therapeutic Strategies Targeting Mitochondrial Dysfunction

Mitochondrial dysfunction plays a central role in the pathogenesis of cardiomyopathy and heart failure, making it an attractive therapeutic target. Several strategies, ranging from pharmacologic interventions to metabolic and lifestyle modulators, have been investigated to restore mitochondrial integrity and function.

Oxidative stress is a hallmark of mitochondrial impairment in failing hearts. Antioxidant supplementation has been extensively tested, with coenzyme Q10 (CoQ10) receiving particular attention. In the randomized, double-blind Q-SYMBIO trial of 420 patients with chronic heart failure, long-term CoQ10 supplementation significantly reduced major adverse cardiovascular events and mortality compared with placebo, suggesting that improving electron transport and reducing oxidative stress can translate into clinical benefit [105].

Another pharmacological strategy involves inhibition of the mPTP, whose opening during ischemia–reperfusion contributes to cell death. Early preclinical studies suggested the protective effects of cyclosporine A [106]. However, large clinical trials failed to confirm these findings. The CIRCUS trial (*n* = 970 anterior STEMI patients undergoing PCI) showed no reduction in all-cause mortality, heart failure hospitalization, or adverse remodeling at one year [107]. Similarly, the CYCLE trial (*n* = 410 STEMI patients) found no benefit of cyclosporine on reperfusion success, infarct size, or left ventricular recovery [108]. Together, these trials underscore the challenges of translating mitochondrial-targeted cardioprotection into clinical efficacy.

Emerging therapeutic strategies aim to correct mitochondrial dysfunction directly. A central focus has been PGC-1α, the master regulator of mitochondrial biogenesis and oxidative metabolism. Knockout studies revealed that PGC-1α deficiency reduces oxidative phosphorylation gene expression, diminishes mitochondrial activity, and impairs the heart’s ability to meet energetic demand [109]. Targeting the broader PGC-1 family (PGC-1α, PGC-1β, PRC) is also promising, as these coactivators coordinate OXPHOS, fatty acid oxidation, and antioxidant defense, and are increasingly recognized as candidate targets for cardiac metabolic therapy [110].

Another innovative approach is mitochondrial transplantation. Preclinical ischemia–reperfusion models show that delivery of healthy mitochondria into injured myocardium enhances ATP production, reduces ROS, and improves cardiomyocyte survival [111]. Systematic reviews confirm protective effects across animal models, though delivery methods and dosing vary [111]. Early human feasibility is encouraging: in pediatric patients with ischemic cardiomyopathy, autologous mitochondrial transplantation improved myocardial bioenergetics and enabled successful weaning from mechanical support in several cases [112]. Collectively, gene-based activation of mitochondrial biogenesis and mitochondrial transfer therapies provide promising avenues to restore cardiac energetics. While still in preclinical and early clinical stages, these strategies underscore the feasibility of directly targeting mitochondria to treat cardiomyopathy and heart failure.

Lifestyle and metabolic interventions are crucial strategies for combating mitochondrial dysfunction in heart failure. Exercise training is the most established non-pharmacological intervention. In a randomized trial, aerobic interval training was superior to moderate continuous training, producing larger improvements in VO2 peak, reverse remodeling, endothelial function, and skeletal muscle mitochondrial capacity in patients with post-infarction heart failure [113]. Caloric restriction mimetics such as spermidine also protect the heart through mitochondrial pathways. Eisenberg et al. demonstrated that oral spermidine reduced cardiac hypertrophy, preserved diastolic function, and enhanced mitophagy and respiration in mice. In humans, a higher dietary intake correlated with reduced blood pressure and cardiovascular events [114]. The metabolic modulation of substrate utilization has been clinically validated. In randomized trials, trimetazidine improved ejection fraction, NYHA class, and quality of life in systolic heart failure. Metabolic data showed reduced resting energy expenditure, consistent with a shift toward glucose oxidation [115]. The most transformative pharmacologic advances are the sodium–glucose cotransporter 2 (SGLT2) inhibitors. In the DAPA-HF trial, dapagliflozin reduced the risk of cardiovascular death or worsening heart failure in patients with HFrEF, independent of diabetes status [116]. The EMPEROR-Reduced trial confirmed that empagliflozin lowered this composite endpoint by 25% and slowed renal decline [117]. Benefits extended to preserved EF: the EMPEROR-Preserved [118] and DELIVER [119] trials both demonstrated significant reductions in cardiovascular death or heart failure hospitalization across HFpEF and HFmrEF populations. Mechanistically, SGLT2 inhibitors modulate myocardial energetics by promoting ketone body utilization, improving mitochondrial respiration, and reducing oxidative stress, thereby enhancing the efficiency of the failing heart [120,121]. A summary of pharmacological, genetic, lifestyle, and metabolic interventions targeting mitochondrial dysfunction is presented in Table 1.

Taken together, exercise, caloric restriction mimetics, substrate modulators, and SGLT2 inhibitors converge on restoring mitochondrial health, positioning mitochondria as both a pathophysiologic driver and a therapeutic target in heart failure. Targeting mitochondrial dysfunction through pharmacologic, genetic, lifestyle, and metabolic interventions highlights mitochondria as both a central driver of cardiomyopathy and a promising therapeutic target in heart failure.

## 6. Conclusions and Future Perspectives

Despite the growing body of evidence linking mitochondrial dysfunction to the pathophysiology of cardiomyopathy and heart failure, translating this knowledge into effective clinical interventions remains a formidable challenge. Mitochondria are intricately integrated into nearly every facet of cardiomyocyte biology, making them both a promising and difficult therapeutic target. Their double-membrane structure, dynamic behavior, and complex interaction with nuclear signaling and cellular metabolism complicate drug delivery, specificity, and efficacy.

One of the main challenges in targeting mitochondria is achieving therapeutic precision without disrupting physiological mitochondrial functions [122]. Unlike conventional drug targets such as receptors or enzymes, mitochondria are not static structures; they constantly undergo structural changes, move within cells, and vary in quantity and function between tissues and even within cardiomyocytes. This plasticity demands tailored therapeutic approaches that can adapt to both temporal and spatial mitochondrial dynamics [123]. Furthermore, mitochondrial-targeted therapies must overcome pharmacokinetic obstacles such as crossing the plasma and mitochondrial membranes. Lipophilic cations (e.g., triphenylphosphonium-based compounds) have been used to deliver bioactive molecules directly into mitochondria, but these approaches may lead to off-target effects and accumulation-related toxicity [124]. Additionally, while numerous compounds (e.g., mPTP inhibitors, antioxidants, electron transport chain modulators) have shown promise in preclinical models, many have failed to demonstrate consistent efficacy in large-scale clinical trials due to differences in mitochondrial phenotype across patient populations and disease stages [125].

Another crucial barrier to clinical translation is the lack of reliable, validated biomarkers of mitochondrial health in cardiovascular disease. The ability to monitor mitochondrial function dynamically and non-invasively is essential for both diagnosis and therapeutic stratification [126]. Currently, most assessments rely on indirect markers such as circulating lactate levels, oxidative stress indicators, or biopsy-derived electron microscopy and respirometry data [127]. However, emerging candidates such as circulating mitochondrial DNA (mtDNA), cardiolipin oxidation products, and mitokines (e.g., FGF21, GDF15) offer new promise. Circulating mtDNA, released during mitochondrial damage or apoptosis, has been associated with inflammation, endothelial dysfunction, and adverse outcomes in heart failure [128]. Similarly, elevated levels of oxidized cardiolipin have been correlated with mitochondrial membrane destabilization and activation of the NLRP3 inflammasome [129]. Advanced imaging modalities, including PET tracers targeting mitochondrial membrane potential (e.g., 18F-TPP+) and hyperpolarized magnetic resonance spectroscopy [130,131], are also being explored as non-invasive techniques to assess mitochondrial metabolism and redox status in vivo. The future clinical utility of these tools lies not only in diagnosis but also in predicting therapeutic responsiveness and disease trajectory.

As the heterogeneity of mitochondrial dysfunction across different cardiomyopathies becomes more apparent, a one-size-fits-all treatment strategy is unlikely to be effective [30]. Instead, the future lies in personalized, phenotype-driven approaches [125]. Genomic, metabolomic, and proteomic profiling could help stratify patients based on specific mitochondrial signatures, such as respiratory chain defects, mitophagy insufficiency, or oxidative stress burden, and guide individualized treatment regimens [132]. Several ongoing clinical trials are exploring mitochondria-targeted therapies in cardiovascular diseases. For example, elamipretide, a peptide that targets cardiolipin to stabilize mitochondrial membranes and improve electron transport efficiency, is under investigation for heart failure with reduced ejection fraction [133]. Another promising agent, bendavia, aims to modulate mitochondrial bioenergetics and reduce ROS production [134]. Trials involving NAD^+^ precursors (e.g., nicotinamideriboside) are also underway, based on their role in mitochondrial biogenesis and sirtuin inactivation [135]. In parallel, lifestyle and metabolic interventions, such as caloric restriction, intermittent fasting, and exercise, are being repurposed and studied for their mitochondria-enhancing properties. These strategies modulate mitochondrial dynamics and may offer low-cost adjuncts to pharmacological therapies [136,137].

As our understanding of mitochondrial biology in cardiac pathology deepens, the future of heart failure management may shift from conventional hemodynamic support to bioenergetic optimization. Yet, to realize this potential, several critical steps must be taken: refining delivery strategies, developing specific and safe modulators of mitochondrial pathways, and establishing robust biomarkers to monitor mitochondrial function longitudinally. Ultimately, mitochondria are not merely passive energy suppliers but central regulators of cardiomyocyte fate and immune–metabolic signaling. Their dysfunction is not a byproduct of disease; it is a driver. Therefore, targeting mitochondrial dysfunction represents not just a therapeutic opportunity, but a conceptual shift in how we understand and treat heart failure.

Continued integration of basic science, translational research, and precision medicine approaches will be essential to transform this opportunity into a clinical reality. If successful, this mitochondria-focused paradigm may unlock new avenues for cardioprotection, improve quality of life, and reduce mortality for millions of patients living with cardiomyopathy and heart failure.

## Figures and Tables

**Table 1:** Summary of therapeutic strategies targeting mitochondrial dysfunction in heart failure.

Therapeutic Strategy	Mechanism/Rationale	Evidence/Key Findings	References
Antioxidant supplementation (Coenzyme Q10)	Improves electron transport, reduces oxidative stress	Q-SYMBIO trial (420 patients): reduced major CV events and mortality	[105]
mPTP inhibition (Cyclosporine A)	Prevents mitochondrial permeability transition pore opening during reperfusion	Preclinical benefit; CIRCUS and CYCLE trials: no clinical efficacy in STEMI patients	[106,107,108]
PGC-1 family activation (PGC-1α, PGC-1β, PRC)	Enhances mitochondrial biogenesis, OXPHOS, fatty acid oxidation, antioxidant defense	Knockout studies: PGC-1α deficiency reduces mitochondrial activity and cardiac energy supply	[109,110]
Mitochondrial transplantation	Delivery of healthy mitochondria to restore ATP production and reduce ROS	Preclinical: improved cardiomyocyte survival; Human feasibility: improved myocardial bioenergetics in pediatric ischemic cardiomyopathy	[111,112]
Exercise training	Enhances mitochondrial capacity, reverse remodeling, endothelial function	RCT: Aerobic interval training > moderate continuous training in post-infarction HF	[113]
Caloric restriction mimetic (Spermidine)	Promotes mitophagy, mitochondrial respiration, and cardioprotection	Animal studies: reduced hypertrophy, preserved diastolic function; Human data: higher intake → lower BP, fewer CV events	[114]
Metabolic modulation (Trimetazidine)	Shifts substrate utilization toward glucose oxidation	RCTs: improved EF, NYHA class, QoL; reduced resting energy expenditure	[115]
SGLT2 inhibitors (Dapagliflozin, Empagliflozin)	Enhance ketone utilization, mitochondrial respiration, and reduce oxidative stress	DAPA-HF, EMPEROR-Reduced, EMPEROR-Preserved, DELIVER trials: reduced CV death and HF hospitalization across HFrEF, HFpEF, HFmrEF	[116,117,118,119,120,121]

## Data Availability

Not applicable.
